# Supplementary figures and images for: The effect of weight loss interventions in truck drivers: Systematic review
Source: PLoS One. 2022 Feb 23;17(2):e0262893. doi: 10.1371/journal.pone.0262893 (PMC8865692; doi:10.1371/journal.pone.0262893)

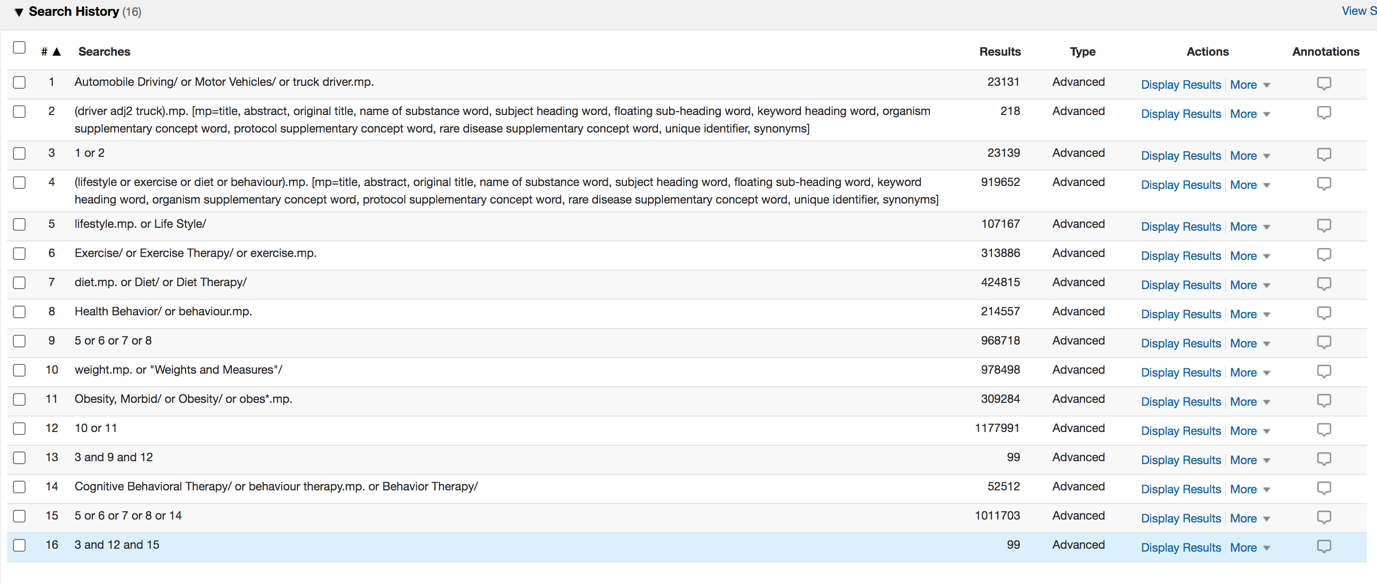

Supplement: S1 Appendix — (TIF) [file pone.0262893.s001.tif]
