# Supplementary material for: The effect of weight loss interventions in truck drivers: Systematic review
Source: PLoS One. 2022 Feb 23;17(2):e0262893. doi: 10.1371/journal.pone.0262893 (PMC8865692; doi:10.1371/journal.pone.0262893)
Supplement: Attachment — Submitted filename: Response to reviewers.docx [file pone.0262893.s004.docx]

28 July 2021

Dear Lisa Susan Wieland,

Thank you for this opportunity to hone this manuscript to the next stage for publication. We have addressed each of the comments from Reviewers and highlight our response below.

**PLOS One – review comments #2, received 9 July 2021.**

- *Reviewer #1: Thank you for the opportunity to review this manuscript again. The authors have clearly done a great deal of valuable work and the review is now much improved. I have a few outstanding points to mention:
- My previous comments with regard to other outcomes such as type 2 diabetes may not have been clear enough. My suggestion was to examine the studies already included in the review and report whether or not the participants’ T2DM improved (if they already had T2DM), or if they developed T2DM (or other long-term conditions associated with obesity) during the follow-up period. Since one of the main reasons weight loss is encouraged in people with obesity is to prevent or improve conditions such as T2DM (i.e. weight in itself is largely a surrogate outcome) therefore I think it is important to capture whether or not these interventions have an effect on weight loss and on other outcomes. I can see from the search strategy that you would not need to go back and search all over again, the issue would be to extract more data from the studies that are already in the review.*

Added outcomes in methods – Page 7:… or impact on long-term health conditions associated with obesity e.g. Type-2 diabetes, or Metabolic Syndrome (MeS), that raises the risk of heart disease.

Added paragraph in results: page 10

Four studies explored additional health risk factors relating to weight i.e. two measured blood glucose levels with readings identified as 95.81 (mg/dl) pre and 110.44 post, both of which were in the normal range (not significant at p=0.46) with no follow-up testing done at 30 months [26], and the other reported 123 (mg/dl) at baseline decreasing to 98 on exit (not significant at p=0.79) [39]. One reported the presence of diabetes (type not stated) with control at 13% at baseline, the intervention group at 12% and blood glucose risk with slight increase across both groups at 6 months which was not significant (p=0.84) [6], and one study reported the presence of Type-2 diabetes as 1% at baseline (no follow-up data) with over two-thirds (71%) having MeS at baseline which decreased in the two intervention groups at 12 months to 62% (in the LIFE group) and to 60% (in the REF group), also not significant (p=0.34) [37].

In the discussion: Page 19/20

None of the four studies that measured incidence of diabetes or MeS, showed a significant reduction [6, 26, 37, 39].

- *The text still has some typos and grammar errors, e.g. subject-verb agreements.*

Changed the following:

Page 1 to: ‘dominated population are exposed’

Page 6: ‘interventions were effective for weight’

Page 7: ‘All eligible studies were discussed in this’

Page 8 ‘Findings have been presented’

Page 21: ‘note that there were additional theoretical’

Page 22: ‘which also impacted the findings’

Page 23 ‘The strengths of this review were the specificity’

Page 23: ‘We discovered there were few high-quality’

*- There are also still some instances of language that could be perceived as judgmental, e.g. ‘obese truck drivers’ instead of ‘truck drivers with obesity’.*

(only one was found and removed page 19 as per comment below)

*- It would be great to see the findings put into some kind of clinical context. For instance, at the moment the abstract reads as if there were some statistically significant differences found in favour of the interventions but it is not clear if those differences are clinically meaningful. It would be very useful for the reader to see some indication of the clinical significance (or not) in the abstract, results and discussion sections.*

Sentence added in the **abstract**

Results: The effect sizes for 5/7 studies were medium to large size (5/7 studies), indicating likely clinical significance.

Conclusion:

Interventions that include a combination of coaching and other resources may provide successful weight reduction for truck drivers and *holds clinical significance in guiding the development of future interventions in this industry*. However, additional trials across varied contexts with larger sample populations are needed.

Main document:

Results: page 10

The majority of the effects observed are medium to large size, indicating likely clinical significance.

Added into discussion page 23:

The clinical significance of these results is important to note, as even a 1-kilogram reduction in weight can reduce the risk of diabetes by 16% [52]. With any reduction in risk of long-term health conditions developing, we are supporting the health and wellbeing of truck drivers. Only 2 studies reported a significant difference with a small effect (so there is the possibility that the effect is not clinically significant), whereas 5 of the differences identified were a medium to large effect, suggesting the differences observed are likely to be clinically significant and meaningful. Using a multi-pronged method of delivery as described in the studies with the most efficacious findings in this review, is more likely to yield effective long-term results, however, many of the studies were small in number with varied effect sizes and additional research is still required.

*- Is there a typo in the following sentence? I am not sure what it is trying to say: “The effect of assignment regarding was the following outcomes (weight, BMI, fat mass, body measurements), converting all to kilograms (kg) and centimeters (cm) for ease of comparison, reporting effect sizes between baseline and reassessment 140 and/or follow-up if provided.”*

Changed – page 7 to “All outcome measurements (weight, BMI, fat mass, body measurements), were converted to kilograms (kg) and centimeters (cm) for ease of comparison, reporting effect sizes between baseline and reassessment and/or follow-up if provided.”

*- Discussion: “This systematic review has identified seven intervention studies conducted over the past 20 years that* *explored the effect of weight loss interventions on obese truck drivers.” I don’t think the latter part of the sentence is completely accurate since the inclusion criteria did not specify that the truck drivers had to have obesity, rather the review focuses on studies of lifestyle interventions for truck drivers in general, regardless of their starting weight. In other words, I suggest removing the word ‘obese’ from this sentence.* Removed the word obese – page 19

*Reviewer #2: The author(s) have sufficiently addressed each of my comments. I have no further comments other than to thank the authors for the opportunity to read and review their work.*

Thank you again for this opportunity, and we hope we have addressed all the remaining concerns and comments.

Kind regards

Dr Elizabeth Pritchard

On behalf of the research team
